# Evaluation of *Bna.SCT* and *Bna.REF1* as Target Genes to Reduce Sinapine in Rapeseed Using a Protoplast‐Based CRISPR RNP Approach

**DOI:** 10.1111/ppl.70905

**Published:** 2026-05-08

**Authors:** Oliver Moss, Xueyuan Li, Selvaraju Kanagarajan, Eu Sheng Wang, Emelie Ivarson, Li‐Hua Zhu

**Affiliations:** ^1^ Department of Plant Breeding Swedish University of Agricultural Sciences Lomma Sweden

**Keywords:** antinutritional factors, CRISPR RNP genome editing, DNA‐free, PEG‐mediated protoplast transfection, rapeseed seedcake, sinapine, transgene‐free mutants

## Abstract

Rapeseed is a major oil crop worldwide, producing both oil and a high amount of protein. However, the use of its seed meal as a protein source for animal feed is limited by antinutritional factors, such as sinapine, which reduces nutrient absorption and affects the palatability. Efforts to reduce sinapine levels through conventional breeding have had limited success. Given the challenges of a changing climate and a growing global population, maximising crop utility, particularly the value of seed meal as a byproduct, is increasingly important. Genetic modification has been successfully used to reduce sinapine in rapeseed, but regulatory restrictions limit its commercial adoption in some regions. CRISPR‐Cas gene editing, which is gaining broader global acceptance, offers a promising alternative to directly produce transgene‐free mutants. In this study, we build on our previous work by generating transgene‐free rapeseed mutants using protoplast‐based CRISPR RNP gene editing. We successfully targeted the *Bna.SCT* and *Bna.REF1* genes with editing efficiencies of 22%–63%, frequently achieving mutations in all four alleles of the target genes in T_2_ plants with a single sgRNA. Seed sinapine content was reduced by up to 38% in *Bna.SCT* mutants and 77% in *Bna.REF1* mutants, with no observed effects on plant growth or development. These findings suggest that *Bna.REF1* is the most effective target for sinapine reduction in transgene‐free mutants among the genes tested in our lab.

## 1 Introduction

Rapeseed ( *Brassica napus* L.) is a major oilseed crop in temperate regions, valued for both its high‐quality oil and its protein‐rich fraction (Cheng et al. 2022). Rapeseed meal is a promising alternative to soybean meal due to its high protein content and well‐balanced amino acid composition (Yoshie‐Stark et al. 2006). However, its broader utilization in animal feed is constrained by the presence of antinutritional compounds, particularly sinapine. This secondary metabolite impairs nutrient digestibility, contributes to bitter flavours, and causes egg taint in the eggs produced by certain poultry breeds (Kozlowska et al. 1990; Qiao et al. 2008; Ward et al. 2009). Efforts to reduce sinapine content through traditional breeding have been limited by the low natural genetic variation within rapeseed germplasm (Zum Felde et al. 2007).

To overcome this limitation, mutagenesis‐based approaches such as Targeting Induced Local Lesions in Genomes (TILLING) have been employed to create low‐sinapine lines. Using TILLING, homozygous mutants for *UDP‐GLUCOSE:SINAPATE GLUCOSYLTRANSFERASE* (*Bna.SGT*) and *REDUCED EPIDERMAL FLUORESCENCE1* (*Bna.REF1*) were generated, achieving sinapine reductions of 57% and 71%, respectively (Emrani et al. 2015). While demonstrating the feasibility of reducing sinapine without transgenic modification, TILLING remains labour‐intensive, requiring extensive mutant screening and backcrossing to eliminate unwanted background mutations.

Since the early 2000s, transgenic approaches have provided further progress in sinapine reduction through RNA interference (RNAi) and antisense RNA technologies. For instance, silencing *Bna.SGT* via RNAi resulted in a 72% reduction (Hüsken et al. 2005), while targeting *Bna.REF1* yielded a 45% reduction (Mittasch et al. 2013). Antisense suppression of *FERULIC ACID 5‐HYDROXYLASE* (*Bna.FAH*) reduced sinapine by 40% (Nair et al. 2000), and RNAi‐mediated silencing of *SINAPOYLGLUCOSE:CHOLINE SINAPOYLTRANSFERASE* (*Bna.SCT*) achieved a 52% reduction. Notably, a sinapine reduction as high as 90% was observed following simultaneous silencing of *Bna.FAH* and *Bna.SCT* (Bhinu et al. 2009). These studies collectively demonstrate that targeted genetic interventions can effectively reduce sinapine accumulation in rapeseed seeds.

Despite these successes, the application of genetically modified (GM) crops remains restricted in many regions, particularly in Europe. This limitation, coupled with the demand for faster crop improvement strategies to address global food security and climate challenges, highlights the need for alternative, non‐transgenic genetic modification methods (Voss‐Fels et al. 2019). New genomic techniques (NGTs), such as CRISPR‐Cas systems, offer precise, efficient, and potentially transgene‐free solutions to crop improvement. CRISPR has been shown to accelerate breeding timelines by up to twofold compared with conventional approaches (May et al. 2023), and recent regulatory developments indicate growing acceptance of NGTs in various regions (European Parliament 2024; Rabuma et al. 2024).

Using our previously established protocol for protoplast regeneration in rapeseed (Li et al. 2021), we recently demonstrated the successful generation of transgene‐free mutants via CRISPR ribonucleoprotein (RNP) mutagenesis targeting *Bna.SGT* (Moss et al. 2025). The resulting mutants exhibited significantly reduced sinapine levels, confirming the potential of DNA‐free CRISPR editing for metabolic trait improvement in rapeseed.

Building on these findings, the present study aims to further investigate the potential of two key genes—*Bna.SCT* and *Bna.REF1—*as single‐gene CRISPR‐Cas targets for reducing sinapine content. *Bna.SCT* encodes SINAPOYLGLUCOSE:CHOLINE SINAPOYLTRANSFERASE, the terminal enzyme in sinapine biosynthesis that catalyses the conversion of sinapoylglucose to sinapine (Milkowski et al. 2004). Due to its seed‐specific expression (Weier et al. 2008), *Bna.SCT* is particularly attractive for targeted mutagenesis, as disruption of its function could lower sinapine accumulation specifically in seeds without affecting upstream phenylpropanoid intermediates essential for plant physiology. There are two *Bna.SCT* loci, *Bna.SCT1* (C genome) and *Bna.SCT2* (A genome) (Weier et al. 2008), simplifying CRISPR targeting due to the low copy number.

Similarly, *Bna.REF1*, which functions two steps upstream of *Bna.SCT* in the phenylpropanoid pathway, has demonstrated potential as a sinapine reduction target. RNAi suppression of *Bna.REF1* resulted in a 58% decrease in sinapine (Mittasch et al. 2013), while mutagenesis yielded a greater reduction of 71% (Emrani et al. 2015). Like *Bna.SCT*, *Bna.REF1* exists as two paralogues, *Bna.REF1_I* (C genome) and *Bna.REF1_II* (A genome), further enhancing its suitability for CRISPR‐mediated editing.

The objective of this study is to assess the efficiency of DNA‐free CRISPR‐Cas mutagenesis targeting *Bna.SCT* and *Bna.REF1* for the generation of transgene‐free, low‐sinapine mutant lines in rapeseed.

## 2 Materials and Methods

### 2.1 Plant Material and Sterilisation

Seeds used in this study were spring rapeseed ( *Brassica napus* L.) cv. Kumily, a doubled haploid, provided by Lantmännen, Sweden. Seeds were surface‐sterilised by gentle shaking in 70% ethanol for 15 min, followed by gentle shaking in 20% kitchen bleach for 15 min. The seeds were then washed in sterile water four times.

### 2.2 In Vitro Seedling Growth and Growth Conditions

Sterilised seeds were grown on germination medium in single‐use sterile plastic boxes. The germination medium contained half‐strength Murashige and Skoog (MS) (Duchefa), 10 g L^−1^ sucrose and 7 g L^−1^ Bacto agar at pH 5.7. The boxes were maintained in a climate chamber, which had a 16‐h photoperiod, with a light intensity of 40 μmol m^−2^ s^−1^ (cool white, fluorescent tubes). The temperatures were 23°C/18°C for day and night, respectively.

### 2.3 Sequencing of *Bna.SCT* and *Bna.REF1* Paralogues

For sequencing the *Bna.SCT* and *Bna.REF1* paralogues, DNA was extracted from the leaves of rapeseed cv. Kumily using the GeneJet Plant Genomic DNA Purification Mini Kit (Thermo Fisher Scientific). PCR was performed using primers (Table 1) designed using the full sequences for *Bna.SCT1* (AM706349), *Bna.SCT2* (AM706350), *Bna.REF1_I* (FN995990) and *Bna.REF1_II* (FN995991) from NCBI. The first 900 bp of each gene was amplified using the pJET1.2 forward sequencing primer to confirm sequence identity with the reference sequences from NCBI. After verifying 100% sequence identity, we used the NCBI reference sequences for CRISPR site prediction and sgRNA design. The PCR product was purified using NucleoSpin Gel and PCR Clean‐up, Mini kit (Macherey‐Nagel) according to the manufacturer's instructions. The purified PCR product was then cloned into the pJET1.2/blunt vector using the CloneJET PCR Cloning Kit (Thermo Scientific). Transformation was conducted using Stellar Competent Cells (Takara Bio) to propagate the recombinant plasmid. Plasmid DNA was subsequently purified from bacterial cultures with the NucleoSpin Plasmid Mini Kit (Macherey‐Nagel), yielding high‐purity plasmid DNA suitable for downstream applications. The recombinant plasmids were then sent to Eurofins (Germany) for Sanger sequencing of the genes.

**TABLE 1:** CRISPR target sequences and primers for gene sequencing and target site amplicon sequencing.

Primer/sgRNA	Sequence 5′‐3′ (sgRNA PAM in bold)	Purpose
SCT F	TTCGCATGCAAGGCTAGTGA	Whole gene amplification
SCT R	CAGAGAGATTCACCATCAATCCATC	Whole gene amplification
SCT amplicon F	GCATGCAAGGCTAGTGACAC	Amplicon sequencing
SCT amplicon R	AGCTGCATCCAGGCCCAC	Amplicon sequencing
REF1 F	TGTCCTCTGAACATAGAGAGAGAGA	Whole gene amplification
REF1 R	AATGGACAAAAGAAAAACGTACTGT	Whole gene amplification
REF1_I amplicon F	TGTCCTCTGAACATAGAGAGAGAGA	Amplicon sequencing
REF1_I amplicon R	GAGAAGAATCTGAGGCAGGGA	Amplicon sequencing
REF1_II amplicon F	CCAAACGTGTAGGCAATCGA	Amplicon sequencing
REF1_II amplicon R	GCCCCATTACTTGATGAATCC	Amplicon sequencing
SCTsgRNA1	**CCG**UUGAGCAUCUUGAUUUUGGU	sgRNA
SCTsgRNA2	**ACC**AAAAUCAAGAUGCUCAACGG	sgRNA
REF1sgRNA1	GAGAACGGUAAAUGCAACGG**AGG**	sgRNA
REF1sgRNA2	TCCUGAUGGCGUGAUCAACG**UGG**	sgRNA

### 2.4 Design of sgRNAs

**FIGURE 1:**
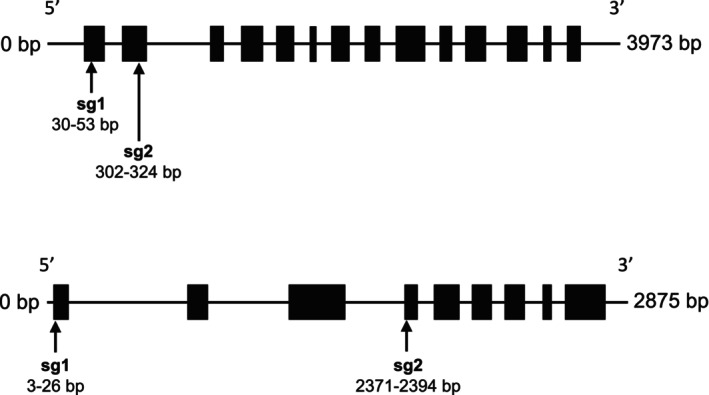
Schematic diagram showing the positions of two sgRNAs (sg1 and sg2) of the *Bna.SCT* gene (top) and *Bna.REF1* gene (bottom) used for knocking out the genes.

Geneious Prime 2024.0.5 was used to predict CRISPR sites and design sgRNAs, and its off‐target checker tool was employed to assess potential off‐target effects. Additionally, the online tool Cas‐OFFinder (Bae et al. 2014) was used to further evaluate off‐target sites (available at http://www.rgenome.net/cas‐offinder/). Two sgRNAs were designed for each gene based on the predicted CRISPR sites (Table 1; Figure 1). These were selected based on high activity scores, absence of off‐target effects, positioning within functional domains of the gene, and location in regions of high sequence homology.

### 2.5 Protoplast Isolation, Transfection and Plant Regeneration

Leaves from 18 to 21‐day old seedlings were used for protoplast isolation using the method described by Li et al. (2021). The transfection was carried out mainly based on the methods reported by Woo et al. (2015) and Moss et al. (2025). In brief, approximately 120,000 isolated and washed protoplasts were re‐suspended in 200 μL freshly prepared MMG solution (0.5 M mannitol, 15 mM MgCl_2_, 4 mM MES) in a 2 mL Eppendorf tube for transfection. The solution was mixed with 20 μL RNP complex solution [4 μL gRNA (0.1 nmol/μL), 4 μL Cas9 (5 μg/μL), and 12 μL H_2_O], and 220 μL freshly prepared PEG‐calcium solution [40% (w/v) PEG 4000, 0.5 M mannitol, 0.1 M CaCl_2_]. The reaction was incubated at room temperature and stopped after 6 min by addition of 1.5 mL W5 and gentle mixing by inversion of the tubes, followed by centrifugation for 3 min at 100*g* and immediate removal of the supernatant. Protoplasts were then embedded in alginate discs and cultured in 6‐well microplates, as described by Li et al. (2021). The embedded protoplasts were cultured for shoot regeneration according to the optimised protocol described in the same study. The in vitro regenerated shoots or putative mutants were rooted on the rooting medium, as described by Li et al. (2021). Once the shoots formed roots, they were transferred to soil pots and grown in the biotron growth chambers, where growth conditions were 21°C/16°C (day/night), 16 h photoperiod with a light intensity of 250 μmol m^−2^ s^−1^ and 60% humidity.

### 2.6 Identification of Mutant Lines

*Naming convention for mutant lines*: Mutant plants were named based on the sgRNA used and their lineage across generations. In identifiers such as **SCT2‐11.5.2**, the numeral immediately following the gene abbreviation (**2**) denotes the sgRNA used for mutagenesis (e.g., **gRNA2**). The subsequent dot‐separated numerals (**11, 5, 2**) denote the plant identifier in successive generations, starting from the initial transfected T_0_ line.

*DNA extraction*: Leaf tissue was taken from in vitro regenerated shoots of putative mutants and crushed with a pipette tip in Phire Dilution Buffer (Thermo Fisher Scientific). The supernatant was used as a template for a PCR reaction using Phusion High‐Fidelity PCR Master Mix (Thermo Fisher Scientific) and gene‐specific primers (Table 1) to amplify the target region containing the sgRNA site. The PCR products were purified using GeneJET Gel Extraction and DNA Cleanup Micro Kit (Thermo Fisher Scientific), and sequenced using Sanger sequencing (Azenta Life Sciences).

*Evaluation of mutant lines in subsequent generations*: T_0_ mutated lines were rooted and grown in the biotron with conditions and management according to Li et al. (2021). The seeds harvested from T_0_ were sown in pots and grown in the biotron. Seeds from T_1_ plants were analysed for sinapine content and the lines with lower sinapine contents were sown to obtain T_2_ seeds. Genotyping was carried out in T_2_ generation. Two mutant plants from each T_2_ line, as well as two WT, were sequenced using amplicon sequencing to acquire accurate sequences from all alleles and to elucidate the type of mutations generated. Genomic DNA was extracted from plants using the GeneJet Plant Genomic DNA Purification Mini Kit (Thermo Fisher Scientific), and PCR was performed using Illumina adapter‐linked primers to amplify the target region, which was then purified using GeneJET Gel Extraction and DNA Cleanup Micro Kit (Thermo Fisher Scientific). The samples were then sent for amplicon sequencing at Eurofins Genomics (Germany).

*Sinapine analysis*: Sinapine analysis was carried out in all generations of mutant lines. Extraction of sinapine was performed from defatted rapeseed meal as described in Moss et al. (2025), with 3 replicates per sample. Sinapine content in the extracted samples was analysed via HPLC as described by Moss et al. (2025) on an Agilent 1260 Series HPLC system, and an Eclipse Plus C18, 3.0 × 100 mm, 3.5 μm column (Agilent). Separation was achieved with an isocratic elution of 10 mM sodium acetate, pH 4.0 (13.5% acetonitrile) at a flow rate of 1 mL/min for 6 min. Sinapine was quantified using a variable wavelength detector (VWD) at a signal wavelength of 330 nm, based on its retention time, and compared to a certified sinapine external standard (ChemFaces).

*Phenotypic observations*: Apart from regular visual observations on growth, flowering time, seed setting, and so forth, 100‐seed weight was measured by weighing 100 seeds on a microbalance.

### 2.7 Statistical Analysis

Data was analysed via one‐way analysis of variance (one‐way ANOVA) and Tukey's honest significance test (Tukey's HSD test) at a significance level of *p* ≤ 0.05. Software: Minitab version 21.4.2 (64‐bit), Minitab LLC.

## 3 Results

### 3.1 Gene Sequencing

The genes were amplified using the primers in Table 1. Sanger sequencing was performed using the pJET1.2 forward sequencing primer (Thermo Scientific), confirming the presence of two paralogues of *Bna.SCT*: *Bna.SCT1* and *Bna.SCT2*, consistent with that by Weier et al. (2008). Similarly, two paralogues of *Bna.REF1*: *Bna.REF1_I* and *Bna.REF1_II* were confirmed, consistent with the sequences reported by Mittasch et al. (2013). The sequences obtained showed 100% identity with the sequences from NCBI.

### 3.2 Mutation Efficiency

For *Bna.SCT*, 35 shoots derived from SCTsgRNA1 and 27 from SCTsgRNA2 were initially screened via Sanger sequencing of PCR products. Six SCTsgRNA1 and 22 SCTsgRNA2 derived shoots were positive for mutations in the sgRNA region, corresponding to a mutation efficiency of 22% and 63%, respectively. For *Bna.REF1*, 16 putative mutants derived from REF1sgRNA1 and 16 from REF1sgRNA2 were screened. Six and nine positive mutants were detected for each sgRNA, corresponding to mutation efficiencies of 38% and 56%, respectively.

### 3.3 Genotyping by Amplicon Sequencing

Positive mutants in T_2_ generation were genotyped using amplicon sequencing to confirm the types of mutations and assess the number of alleles carrying the mutation.

**FIGURE 2:**
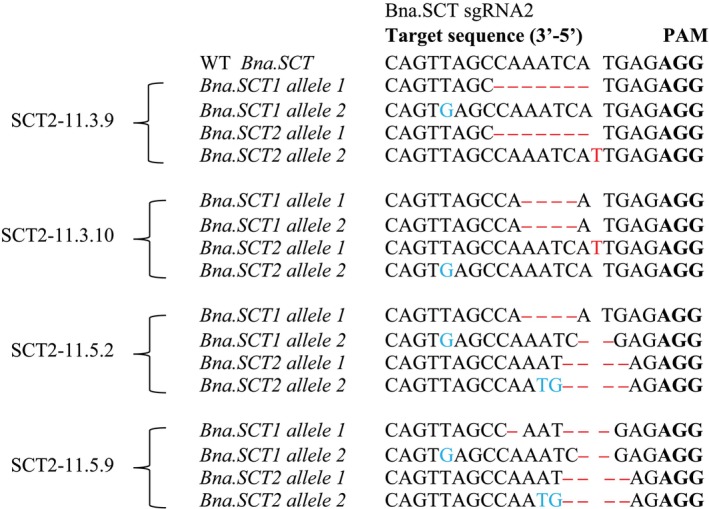
Types of mutations in *Bna.SCT* detected in T_2_ rapeseed mutants derived from SCTsgRNA2 in comparison with wild type (WT), determined by amplicon sequencing. Mutations are indicated by red letters (insertions), ‘–’ (deletions), or blue letters (substitutions). PAM sites are highlighted in bold letters. SCT2 refers sgRNA2 and numbers after the dash denote plant numbers across the T_0_–T_2_ generations.

For *Bna.SCT*, two out of the 12 plants from both T_2_ mutant lines were genotyped. Both SCT2.11.3 lines had mutations on all four alleles of *Bna.SCT*; however, one out of the four alleles contained a silent mutation (SCT2.11.3.9 *Bna.SCT1* allele 2, SCT2.11.3.10 *Bna.SCT2* allele 2), meaning that three out of four alleles were mutated (Figure 2). In contrast, both SCT2.11.5 lines carried meaningful mutations (stop codon or frame shift) on all four alleles.

**FIGURE 3:**
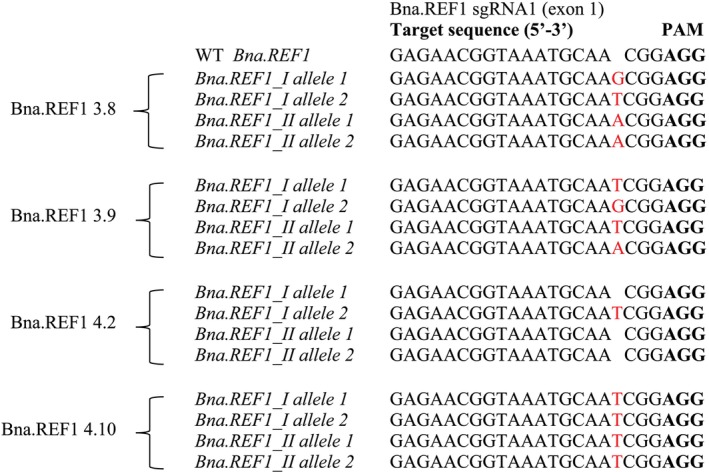
Types of mutations in the *REF1*gene detected in T_1_ rapeseed REF1mutants in comparison with wild type (WT), determined by amplicon sequencing. Mutations are indicated by red letters (insertions), PAM sites are highlighted in bold letters. REF1 refers to sgRNA1 and numbers after the dash denote plant numbers in the T_0_ and T_1_ generations.

**FIGURE 4:**
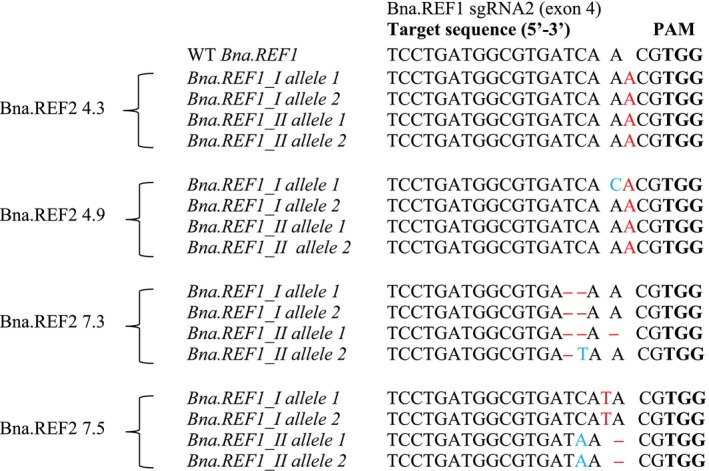
Types of mutations in *Bna.REF2* detected in T_1_ rapeseed REF1sgRNA2 mutants in comparison with wild type (WT), determined by amplicon sequencing. Mutations are indicated by red letters (insertions), ‘–’ (deletions), or blue letters (substitutions). PAM sites are highlighted in bold letters. REF2 refers sgRNA2 and numbers after the dash denote plant numbers across the T_0_–T_2_ generations.

For *Bna.REF1*, two out of the 12 plants of all T_1_ mutant lines were genotyped. The results showed that all plants were mutated in both alleles of both paralogues of *Bna.REF1* apart from plant REF1.4.2, which was mutated in one out of four alleles (Figures 3 and 4).

### 3.4 Sinapine Content

For *Bna.SCT* mutants in T_0_ generation, three plants derived from SCTsgRNA1 and three derived from SCTsgRNA2 were grown in the biotron for phenotyping. The sinapine analysis results for the T_0_ mutants are presented in Figure S1. The seed sinapine content in most mutant lines was similar to that of WT, except line SCT2.11, which showed slightly reduced sinapine content, and line SCT2.12 with an increased sinapine content, possibly due to not all alleles of *Bna.SCT* being mutated.

**FIGURE 5:**
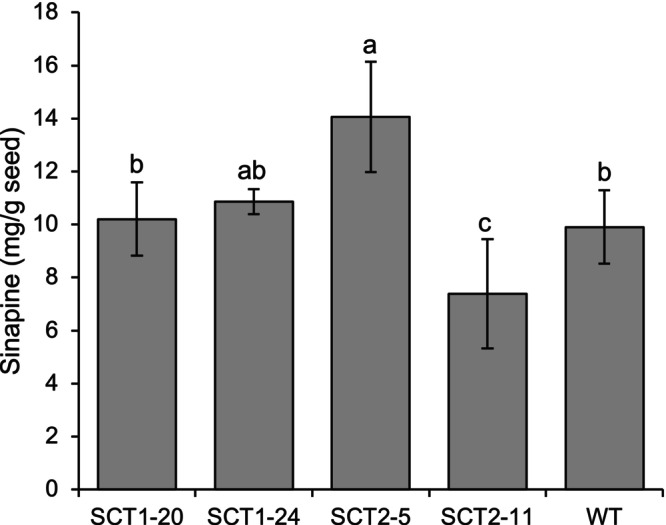
Mean seed sinapine content in T_1_ *Bna.SCT* knockout rapeseed lines compared with wild type (WT), based on Figure S2. Different letters above the bars represent significant differences at *p* ≤ 0.05 with Tukey's HSD test. Error bars represent ± SD (*n* = 5 individual plants). SCT1 and SCT2 refer to sgRNA1 and sgRNA2, respectively. Numbers after the dash denote plant numbers in the T_1_ generation.

Two plants derived from sgRNA1 (SCT1.20 and SCT1.24) and two plants derived from sgRNA2 (SCT2.5 and SCT2.11) were selected for growth in the next generation (T_1_). Five seeds from each line were grown in the biotron. The average sinapine level in line SCT2‐11 showed a significant reduction compared to WT, while a significant increase in sinapine level in line SCT2‐5 was observed (Figure 5). When looking at the individual plant level, only two plants from the line SCT2.11 had clearly reduced sinapine contents compared to WT, while the rest of the plants in different lines had little difference, or even increased sinapine levels (Figure S2).

**FIGURE 6:**
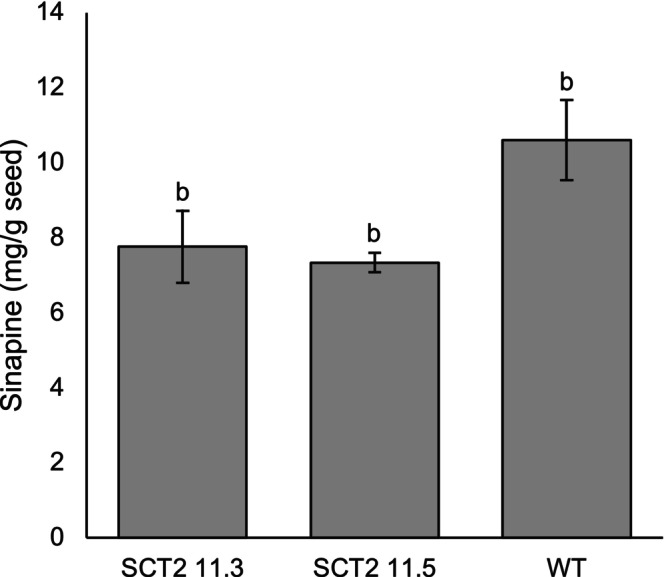
Mean seed sinapine content in T_2_ *Bna.SCT* knockout rapeseed lines compared with wild type (WT), based on Figure S3. Different letters above the bars represent significant differences at *p* ≤ 0.05 with Tukey's HSD test. Error bars represent ± SD (*n* = 12 individual plants). SCT2 refers to sgRNA2 and numbers after the dash denote plant numbers in the T_0_ and T_1_ generations.

Twelve seeds from the two T_1_ lines 3 and 5 with the lowest sinapine contents, SCT2.11.3 and SCT2.11.5, were planted for the next generation (T_2_). The average sinapine reduction for SCT2.11.3 and SCT2.11.5 was 27% and 31%, respectively (Figure 6). The individual mutant with the lowest sinapine was SCT2.11.3.10, which had 6.6 mg/g sinapine in the seed, or a reduction of 38% (Figure S3).

**FIGURE 7:**
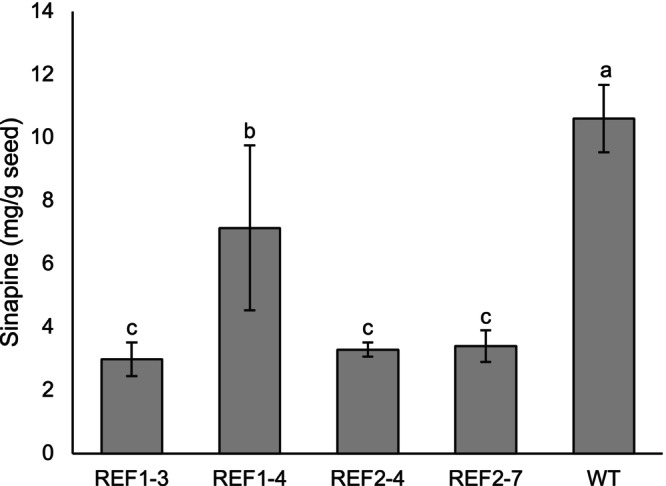
Mean seed sinapine content in T_1_ *Bna.REF1* knockout rapeseed lines compared with wild type (WT), based on Figure S5. Different letters above the bars represent significant differences at *p* < 0.05 with Tukey's HSD test. Error bars represent ± SD (*n* = 12 individual plants). REF1 and REF2 refer to sgRNA1 and sgRNA2, respectively. Numbers after the dash indicate plant numbers in the T_1_ generation.

For *Bna.REF1* mutants in T_0_ generation, four positive mutants derived from REF1sgRNA1 and four from REF1sgRNA2 were analysed for seed sinapine content (Figure S4). Two plants derived from REF1sgRNA1 and two plants derived from REF1sgRNA2 were selected for further evaluation in the T_1_ generation (REF1.3, REF1.4, REF2.4, REF2.7), based on their reduced sinapine levels. The seed sinapine levels in individual plants in each mutant line showed a consistent sinapine reduction for all lines, apart from REF1.4, due to the line segregating (Figure S5). The average sinapine content in all lines had a significant reduction in comparison to WT (Figure 7).

The T_1_ *Bna.REF1* mutant line with the lowest sinapine was REF1.1.3, with an average sinapine level of 2.9 mg/g, or a reduction of 73%. The individual mutant with the lowest sinapine had a value of 2.44 mg/g, corresponding to a 77% reduction.

Line REF1.4 had a mixture of plants with reduced and normal sinapine levels. Genotyping of one of the low and one of the normal sinapine plants (REF1.4.10 and REF1.4.2, respectively) within this line showed that the low sinapine plant carried knockout mutations in all four alleles, whereas the normal sinapine plant carried a knockout mutation in one out of four alleles (Figure 3). This suggests that line REF1.4 is segregating.

#### 3.4.1 Phenotypic Observation

**FIGURE 8:**
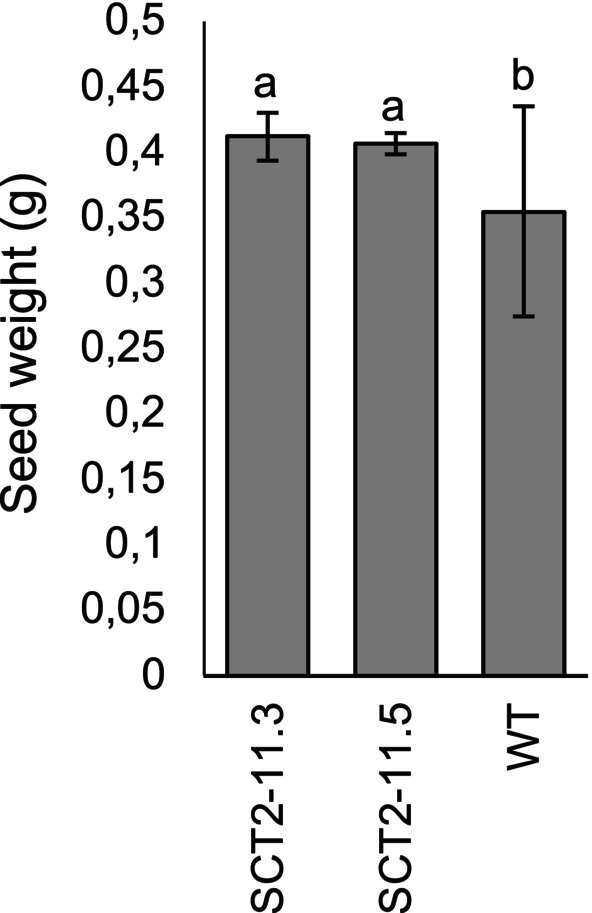
100‐seed weight of *Bna.SCT* mutants in the T_2_ generation and wild type (WT) of rapeseed. Different letters above the bars represent significant differences at *p* ≤ 0.05 with Tukey's HSD test. Error bars represent ± SD (*n* = 12 individual plants). SCT2 refers to sgRNA2. Numbers after the dash denote plant numbers in the T_1_ and T_2_ generations.

**FIGURE 9:**
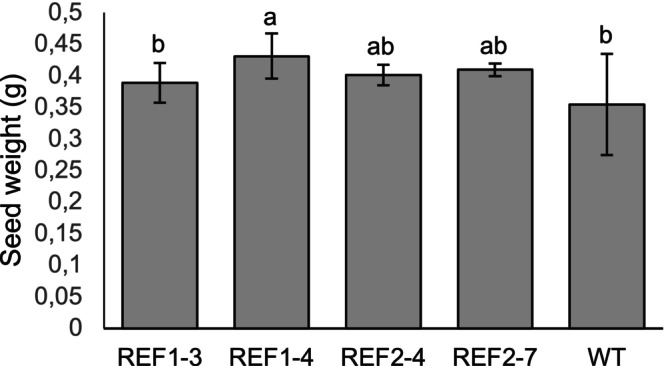
100‐seed weight of *Bna.REF1* mutants in the T_1_ generation and wild type (WT) of rapeseed. Different letters above the bars represent significant differences at *p* ≤ 0.05 with Tukey's HSD test. Error bars represent ± SD (*n* = 12 individual plants). REF1 and REF2 refer to sgRNA1 and sgRNA2, respectively. Numbers after the dash denote plant numbers in the T_1_ generation.

The *Bna.SCT* lines exhibited normal growth comparable to the WT, but the 100‐seed weight of the *Bna.SCT* lines was greater than those of WT plants (Figure 8). Similarly, all *Bna.REF1* lines displayed growth and flowering times consistent with WT. While most *Bna.REF1* lines had 100‐seed weights similar to WT, seeds from REF1‐4 were notably heavier (Figure 9).

## 4 Discussion

In this study, we built upon our previous work in Moss et al. (2025), where the protoplast regeneration protocol adapted from Li et al. (2021) was used for to generate transgene‐free, low‐sinapine mutants of rapeseed by targeting *Bna.SGT*. The current study extends the work of Moss et al. (2025) by evaluating two additional target genes involved in sinapine biosynthesis for CRISPR‐Cas9 editing, namely *Bna.SCT* and *Bna.REF1*, whilst further demonstrating the relative ease of generating transgene‐free mutants of rapeseed. This kind of research is particularly important due to the need for precise, faster breeding methods whilst addressing the drawbacks of other breeding techniques. Regulatory concerns and public perception limit the commercial sale of transgenic crops in Europe, and TILLING comes with practical drawbacks. Crops developed through NGTs, such as CRISPR‐Cas9, are expected to gain market approval in the near future (European Parliament 2024).

The presence of two *Bna.SCT* genes, *Bna.SCT1* and *Bna.SCT2* in rapeseed cv. Kumily, confirmed by sequencing, is consistent with the findings of Weier et al. (2008). Similarly, sequencing confirmed the presence of two *Bna.REF1* paralogues, *Bna.REF1_I* and *Bna.REF1_II*, in agreement with Mittasch et al. (2013).

We demonstrated editing efficiencies of 22% and 63% for SCTsgRNA1 and SCTsgRNA2, and 38% and 56% for REF1sgRNA1 and REF1sgRNA2, respectively. These results further highlight the high editing efficiencies achievable for rapeseed through using our protoplast‐based CRISPR RNP editing approach. It should be borne in mind that protoplast regeneration capacity can be genotype‐dependent. It is thus possible that the efficient protocol established in this study could work directly or may need some modifications in culture media for protoplast regeneration when switching to a new genotype.

The T_0_ *Bna.SCT* mutants had mixed sinapine levels, mostly with minimal changes to the sinapine content, which is likely because not all alleles of the target genes were mutated. In the T_1_ generation, some lines showed clear reduced sinapine content and these were selected for further evaluation in T_2_. The average sinapine reduction for the T_2_ lines SCT2.11.3 and SCT2.11.5 was 27% and 31%, respectively, and a relatively consistent sinapine reduction can be observed across individuals within the lines, particularly in SCT2.11.5. These results show that knocking out *Bna.SCT* influences the accumulation of sinapine in rapeseed seeds, but not to the same extent as other target genes, such as *Bna.REF1* in this study, or *Bna.SGT* in our previous study (Moss et al. 2025). One of the main reasons we chose *Bna.SCT* as a CRISPR target was its position at the end of the phenylpropanoid pathway, making it less likely to disturb the rest of the pathway, causing negative consequences for the plant. However, it is possible that *Bna.SCT's* position in the sinapine biosynthetic pathway makes it less efficient at preventing sinapine production.

Plants mutated with meaningful mutations in three out of four *Bna.SCT* alleles performed equally to those with meaningful mutations in all 4 alleles regarding sinapine content, indicating that a single functional *Bna.SCT* allele may not significantly contribute to sinapine accumulation. This is in contrast to the *Bna.SGT.a* mutants, in which one unmutated allele could lead to unchanged sinapine levels compared to WT (Moss et al. 2025).

In this study, *Bna.REF1* lines exhibited a significant reduction in sinapine, which was already evident in the T_0_ generation. The T_1_ line with the highest reduction in sinapine was REF1.1.3, with a sinapine content of 2.9 mg/g, representing a 73% reduction. The individual mutant with the lowest sinapine content recorded a value of 2.44 mg/g, corresponding to a 77% reduction. These reductions are comparable to those reported by Emrani et al. (2015), who achieved a 71% reduction using EMS mutagenesis, and notably higher than the 58% reduction observed in transgenic *Bna.REF1* mutants by Mittasch et al. (2013). Additionally, our results showed that *Bna.REF1* lines with mutations in only one of the four *Bna.REF1* alleles exhibited little or no reduction in sinapine. This result is consistent with the findings of Harloff et al. (2012) and Emrani et al. (2015), who reported that knocking out one gene copy, affecting both of its alleles, was insufficient to lower sinapine levels.

Regarding phenotypic traits of the edited lines with significantly reduced sinapine contents, we did not observe any differences in the health or well‐being of plants in the T_1_ generation under controlled conditions in the biotron. Further studies on the growth performance of those lines need to be carried out in field trials under natural conditions in order to ensure that knocking out *Bna.REF1* does not negatively impact the plants' well‐being or agricultural properties. Previous research on transgenic *Bna.REF1* knockouts did not evaluate the growth characteristics of the mutants (Mittasch et al. 2013). Nonetheless, it has been observed that Arabidopsis *At.REF1* mutants showed no detectable changes in these factors compared to wild type (Nair et al. 2000). Similarly, Emrani et al. (2015) noted that their *Bna.REF1* EMS mutants exhibited no differences in important growth characteristics compared to EMS controls, further supporting the evidence that *Bna.REF1* knockouts do not cause adverse growth effects.

One concern in reducing sinapine in rapeseed is its role in protecting the plant from UV‐B radiation. Studies on *Bna.SGT* mutants showed that the reduction in sinapine and sinapate esters in rapeseed caused by the silencing of *Bna.SGT* had no negative effect on seed germination, seedling development, or response to UV‐B radiation (Hettwer et al. 2016). These findings may indicate that reductions in sinapine content achieved through *Bna.SCT* and *Bna.REF1* knockouts are unlikely to compromise the plant's ability to withstand UV‐B radiation. However, experimental studies are needed to prove this.

Among the three genes knocked out by CRISPR RNP for reducing sinapine content in the seeds of rapeseed carried out in our lab, it appeared that *Bna.REF1* proved to be the most effective at reducing sinapine, with a 77% reduction in the lowest individual plant, followed by *Bna.SGT* (49%) (Moss et al. 2025), while *Bna.SCT* showed the least sinapine reduction (38%). Further testing on phenotypic traits, plant health, and metabolism would be necessary to make a comprehensive evaluation of these mutant lines. We believe it is possible to further reduce sinapine content by further screening, as there is variation in sinapine content within mutant plants mutated in the same target gene. The most efficient way for achieving even lower sinapine levels is likely by simultaneously targeting multiple target genes.

In conclusion, following from our previous work (Moss et al. 2025), we have further demonstrated the feasibility of rapidly and efficiently generating transgene‐free mutants via protoplast‐based CRISPR RNP gene editing using the protocol developed in our lab (Li et al. 2021). This approach offers a means to overcome the drawbacks of existing breeding and biotechnological techniques.

## Author Contributions

L.H.Z. led the project and edited the draft and final version. O.M. performed experiments and analyzed data, wrote the draft and edited the final version. X.L. performed experiments. E.S.W. performed sinapine and statistical analysis. E.I. and S.K. participated in the experimental design and reviewed the final version. All authors read and commented on the previous version and agreed with the final version of the manuscript.

## Funding

This study was mainly supported by (1) SLU Grogrund‐Centre for Breeding of Food Crops, (2) Trees and Crops for the Future (TC4F), SLU strategic research environment as well as The Royal Physiographic Society of Lund, which were provided to LHZ.

## Disclosure

No AI is used in this manuscript.

## Supporting information


**Figure S1:** Seed sinapine content in T_0_ *Bna.SCT* mutants compared with wild type (WT) of rapeseed. SCT1 and SCT2 denote sgRNA1 and sgRNA2, respectively and numbers after the dash donate mutant lines.
**Figure S2:** Seed sinapine content in T_1_ *Bna.SCT* mutants compared with wild type (WT) of rapeseed. SCT1 and SCT2 denote sgRNA1 and sgRNA2, respectively. The numbers after the dash denote mutant line numbers, while the numbers after the dash denote plant numbers in mutant lines in the T_1_ generation.
**Figure S3:** Seed sinapine content in two T_2_ *Bna.SCT* mutants and wild type (WT) of rapeseed. SCT1 and SCT2 denote sgRNA1 and sgRNA2, respectively. The number 11 after the dash denote mutant line number in T_1_. The numbers 3 and 5 after the first dots denote the line numbers in T_2_ and the last numbers after the second dots donate the plant numbers in the mutant lines 3 and 5, respectively.
**Figure S4:** Sinapine content in the seeds of rapeseed *Bna.REF1* mutants and WT in the T_0_ generation.
**Figure S5:** Sinapine content in the seeds of rapeseed *Bna.REF1* mutants and WT in the T_1_ generation.

## Data Availability

All the relevant data is available within the manuscript.
