# Supplementary material for: Evaluation of Bna.SCT and Bna.REF1 as Target Genes to Reduce Sinapine in Rapeseed Using a Protoplast‐Based CRISPR RNP Approach
Source: Physiol Plant. 2026 May 8;178:e70905. doi: 10.1111/ppl.70905 (PMC13155187; doi:10.1111/ppl.70905)
Supplement: Supplementary file 1 — Figure S1: Seed sinapine content in T0 Bna.SCT mutants compared with wild type (WT) of rapeseed. SCT1 and SCT2 denote sgRNA1 and sgRNA2, respectively and numbers after the dash donate mutant lines. Figure S2: Seed sinapine content in T1 Bna.SCT mutants compared with wild type (WT) of rapeseed. SCT1 and SCT2 denote sgRNA1 and sgRNA2, respectively. The numbers after the dash denote mutant line numbers, while the numbers after the dash denote plant numbers in mutant lines in the T1 generation. Figure S3: Seed sinapine content in two T2 Bna.SCT mutants and wild type (WT) of rapeseed. SCT1 and SCT2 denote sgRNA1 and sgRNA2, respectively. The number 11 after the dash denote mutant line number in T1. The numbers 3 and 5 after the first dots denote the line numbers in T2 and the last numbers after the second dots donate the plant numbers in the mutant lines 3 and 5, respectively. Figure S4: Sinapine content in the seeds of rapeseed Bna.REF1 mutants and WT in the T0 generation. Figure S5: Sinapine content in the seeds of rapeseed Bna.REF1 mutants and WT in the T1 generation. [file PPL-178-e70905-s001.pdf]

Supporting information

Evaluation of *Bna.SCT* and *Bna.REF1* as Target Genes to Reduce Sinapine in Rapeseed Using a Protoplast-based CRISPR RNP Approach

Oliver Moss, Xueyuan Li, Selvaraju Kanagarajan, Eu Sheng Wang, Emelie Ivarson, Li-Hua Zhu

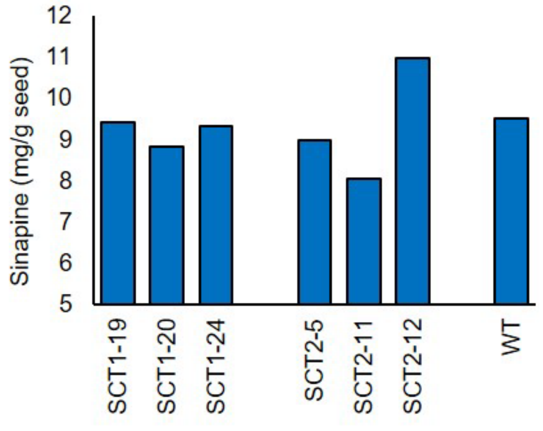

Figure S1. Sinapine content in the seeds of rapeseed *Bna.SCT* mutants and WT in the T_0 generation.

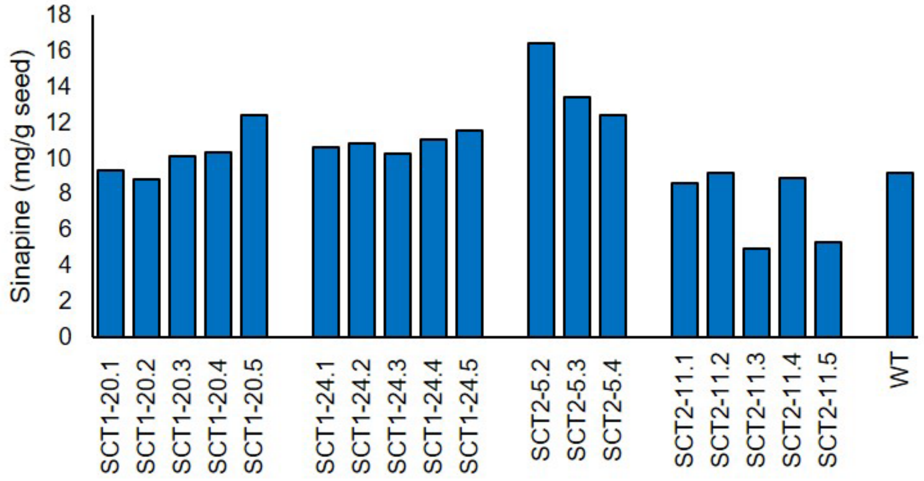

Figure S2. Sinapine content in the seeds of rapeseed *Bna.SCT* mutants and WT in the T_1 generation.

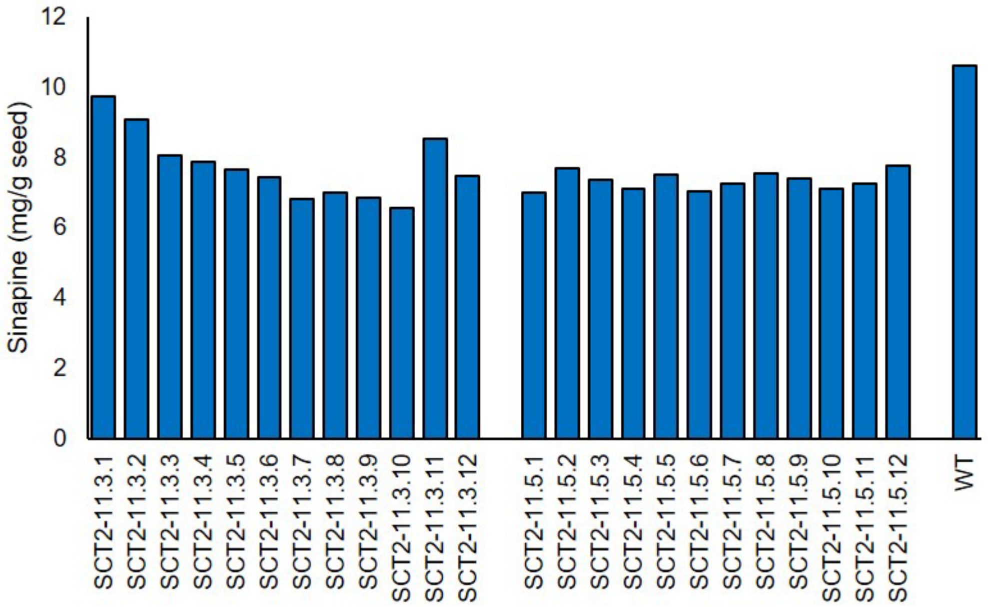

Figure S3. Sinapine content in the seeds of rapeseed *Bna.SCT* mutants and WT in the T_2 generation.

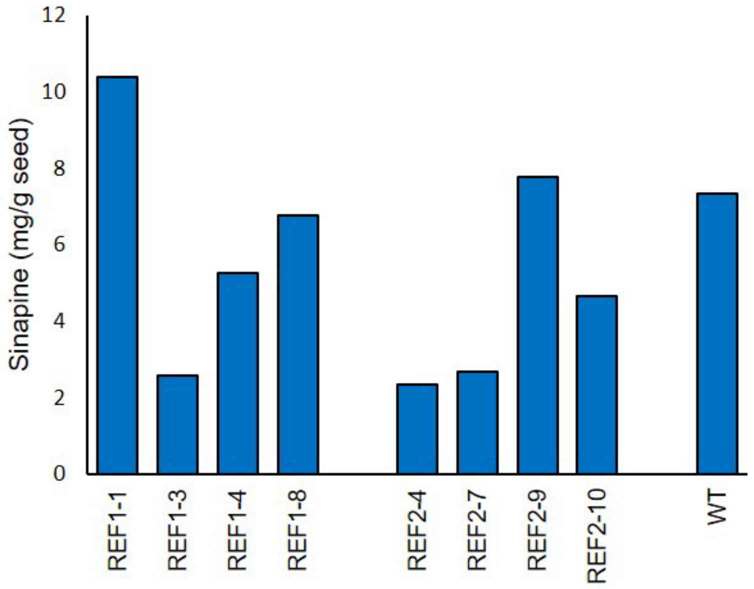

Figure S4. Sinapine content in the seeds of rapeseed *Bna.REF1* mutants and WT in the T_0 generation.

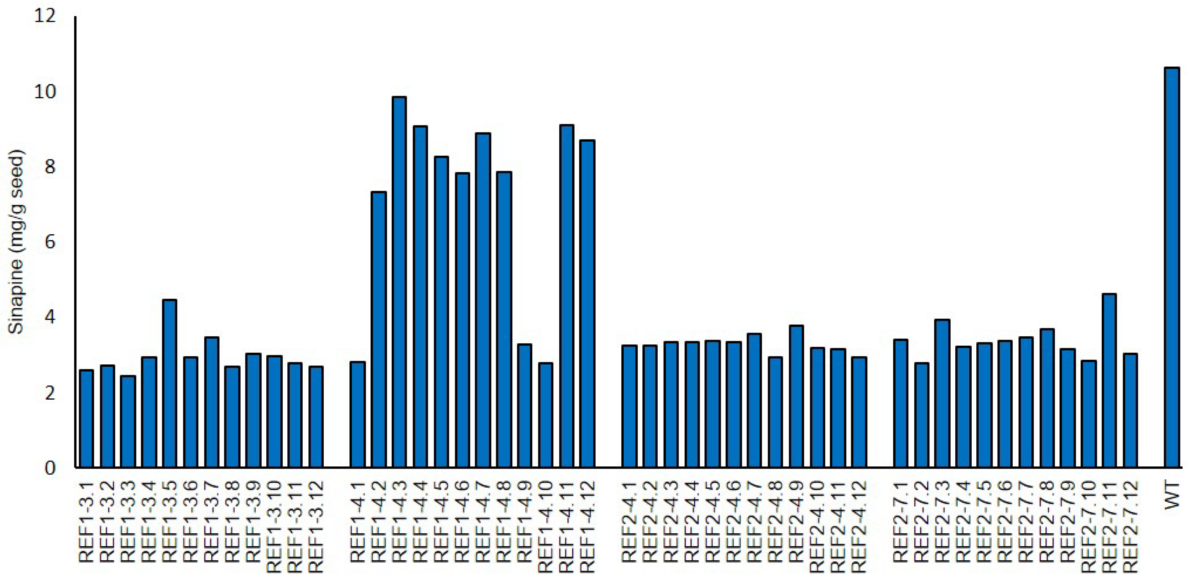

Figure S5. Sinapine content in the seeds of rapeseed *Bna.REF1* mutants and WT in the T_1 generation.
